# Mesenchymal stem cell-derived extracellular vesicles for cell-free therapy of ocular diseases

**DOI:** 10.20517/evcna.2022.08

**Published:** 2022-04-24

**Authors:** Xiaoling Liu, Liang Hu, Fei Liu

**Affiliations:** ^1^Eye Hospital, School of Ophthalmology and Optometry, School of Biomedical Engineering, Wenzhou Medical University, Wenzhou 325000, Zhejiang, China.; ^2^Wenzhou Institute, University of Chinese Academy of Science, Wenzhou 325000, Zhejiang, China.

**Keywords:** Mesenchymal stem cells, extracellular vesicles, ophthalmic diseases, therapy

## Abstract

Mesenchymal stem cells-derived extracellular vesicles (MSC-EVs) have noticeably attracted clinicians’ attention in treating ocular diseases. As the paracrine factor of MSCs and an alternative for cell-free therapies, MSC-EVs can be conveniently dropped over the ocular surface or diffused through the retina upon intravitreal injection, without increasing the risks of cellular rejection and tumor formation. For clinical translation, a standardized and scalable production, as well as reprogramming the MSC-EVs, are highly encouraged. This review aims to assess the potential approaches for EV production and functional modification, in addition to summarizing the worldwide clinical trials initiated for various physiological systems and the specific biochemical effects of MSC-EVs on the therapy of eye diseases. Recent advances in the therapy of ocular diseases based on MSC-EVs are reviewed, and the associated challenges and prospects are discussed as well.

## INTRODUCTION

Mesenchymal stem cells (MSCs) are a heterogeneous population of stromal stem cells that can be isolated from various tissues, including bone marrow, adipose tissue, umbilical cords, and even urine^[1]^. Recently, MSCs have been extensively recognized as an experimental and therapeutic tool spanning from physiological regulation to organ remodeling, due to their superiority in low antigenicity and tumorigenicity^[2-3]^. When the term “Mesenchymal Stem Cell” was searched on ClinicalTrials.gov, more than 1000 clinical trials could be retrieved, which were directly associated with various diseases, suggesting the great potential of the mentioned term in regenerative medicine.

**Figure 1:**
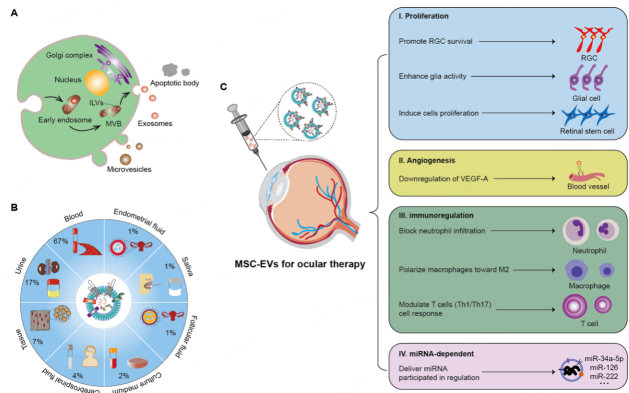
Illustration of biogenesis of EVs and MSC-EVs for ocular therapy. (A) EVs mainly include microvesicles, apoptotic bodies, and exosomes based on their biogenesis. (B) EV sources and the proportion of EV sources derived from 143 clinical trials [www.clinicaltrials.gov (Accessed: August 2021)]. (C) MSC-EVs, which are used for ocular therapy, are associated with the mechanisms of cell proliferation, angiogenesis, immunoregulation, and miRNA-dependent regulation. EVs: Extracellular vesicles; MSC-EVs: mesenchymal stem cells-derived EVs; ILVs: intraluminal vesicles; MVB: multivesicular body; RGC: retinal ganglion cells; VEGF-A: vascular endothelial growth factor A; M2: macrophage of type 2; T cells: thymus-dependent lymphocyte; Th cells: helper T cells; miRNA: microRNA.

The roles of MSCs may include straight differentiation into target cells to replace injured tissues and generate various bioactive substances including extracellular vesicles (EVs), especially nano-sized exosomes^[4,5]^. EVs are generally classified as exosomes (30-150 nm) formed inwardly during the maturation of multiple vesicle endosomes^[6-7]^, microvesicles (50-1000 nm) directly shed from plasma membrane, and apoptotic bodies (1000-5000 nm) released by dying cells^[8,9] ^[Figure 1A]. However, due to the overlapping size range and the lack of specific markers, current “exosome” preparations are a mixture of EVs with undefined biogenesis origin and undetermined purity. According to the MISEV2018 position paper from ISEV^[10]^, in this review, we use the term “MSC-EVs” to describe the MSC-derived exosomal preparations. EVs are released from living cells and can be found in almost all body fluids, including blood, urine, breast milk, tears, saliva, vitreous fluid, and aqueous humor^[11-14]^. The top two most studied body fluids are still blood and urine observed in 143 clinical trials [www.clinicaltrials.gov (Accessed: August 2021)] [Figure 1B]. These nanoparticles carry plenty of bioactive molecules, such as proteins, lipids, RNAs [messenger RNAs (mRNAs), circular RNAs (circRNAs), small RNAs (sRNAs), long non-coding RNAs (lncRNAs)], and DNAs [genomic DNA (gDNA), complementary DNA (cDNA), and mitochondrial DNA (mDNA)]^[15-19]^ that are delivered to recipient cells mediating intercellular response. Compared with MSCs, EV-based therapeutics have shown unique advantages, including cell-free therapy, large-scale EV production, low immunogenicity, and high bioavailability, making these vesicles possible drugs in treating various diseases (e.g., eye diseases).

The human eye has a localized array of surface molecules and cytokines, and it is a sensory organ that reacts with visible light and enables us to use visual information for various purposes^[20-22]^. The intercellular signaling pathway is critical to maintaining the homeostasis of the intraocular microenvironment. EVs from both non-immune and immune cells play important roles in immune regulation^[23]^. At present, for the visual system, researchers have mainly concentrated on the application of EV-based therapeutics in a variety of ocular diseases, such as physical injuries, immune-related diseases, and other eye diseases. These nanoparticles migrate to injured or inflammatory sites, releasing genetic materials or proteins to repair damage by participating in signaling pathways^[24-29]^. As a new model of cell-free therapy, EVs have been evaluated in preliminary clinical trials and have shown great efficacy^[30]^. In addition, EV-associated products have also been applied in the treatment of ocular diseases^[31,32]^. With the continuous exploration of the physical and chemical properties and functions of the EVs, these naturally occurring nanoparticles have been feasibly applied to clinical medicine.

The present review aims to assess the biological characteristics of MSC-EVs and consider novel methods for EV isolation. More importantly, the translational application of MSC-EVs in eye diseases and the current challenges are discussed.

### MSC-EVs

Secretion of the cells in the form of EVs was traditionally considered as unimportant waste material, cellular “garbage bags”, or dust particles, while it was later found that these nano-vesicles are vital messengers and participate in diverse physiological and pathological processes, such as bone tissue regeneration^[33]^, tumor defense^[34]^, nerve signal transmission^[35]^, endothelial cell migration^[36]^, and immune tolerance^[37]^, as summarized in Table 1. The paracrine effect of MSCs was first described by Haynesworth *et al.*^[51]^, who reported the synthesis and secretion of various growth factors, chemokines, and cytokines by MSCs. In 2009, Bruno *et al.*^[52]^ demonstrated that microvesicles derived from MSCs may activate a proliferative program in surviving tubular cells after injury via a horizontal transfer of mRNAs. MSC-EVs were first fractionated with a particle size of 55-65 nm by high-performance liquid chromatography. In total, 857 related proteins and 151 microRNAs (miRNAs) of MSC origin have been detected by mass spectrometry, antibody array technology, and microarray analysis^[53,54]^. Besides, among the functional elements of these EVs, the roles of miRNAs in EV-based therapeutics have been widely investigated^[55-57]^. We mainly summarize several mechanisms achieved by recent studies, including proliferation, angiogenesis, immunoregulation, and miRNA-dependent activity [Figure 1C].

**Table 1:** Summary of the mechanisms and applications of the MSC-derived EV therapies

**Application**	**Disease model**	**EV source**	**Dose**	**Effector molecule**
Tissue regeneration	Nephrectomy	UMSCs	10 μg	Proteins: ANG-1, etc^[38]^
	Renal ischemia reperfusion	hP-MSCs	100 μg	miRNA let-7a-5p^[39]^
	Calvarial bone defect	BMSCs	5 × 10^8^ particles	Protein: BMP2^[40]^
Tumor defense	Metastatic lung nodules	AD-MSCs	2.32 × 10^9^ particles	miR-101^[41]^
	Gastric cancer	UMSCs	64 μg	Protein: L-PGDS^[42]^
	LC	BMSCs	50 μg	Protein: caspase 3^[43]^
Nerve injury	Sciatic nerve transection	UMSCs	100 μg	Protein: IL-10, etc^[44]^
	Brain injury	BMSCs	200 μL (unknown)	miR-140-5p^[45]^
	AD	BMSCs	30 μg	miR-29c-3p^[46]^
Endothelial cell migration	Angiogenesis	OM-MSCs	50 μg	miR-612^[47]^
	Myocardial ischemia	DPSC	3.5 × 10^10^ particles	miR-4732-3p^[48]^
immunoregulation	Knee osteoarthritis	SMSC	5 μL (unknown)	miR-31^[49]^
	Chronic asthma	UMSCs	40 μg	miR-146a-5p^[50]^

EV: Extracellular vesicle; UMSCs: Umbilical cord-derived mesenchymal stem cells; ANG-1: angiopoietin-1; hP-MSCs: human placenta-derived MSCs; BMP2: bone morphogenetic protein 2; AD-MSCs: adipose tissue-derived mesenchymal stromal cells; L-PGDS: lipocalin-type prostaglandin D2 synthase; LC: lung carcinoma; AD: Alzheimer’s disease; OM-MSCs: olfactory mucosa MSCs; DPSC: dental pulp-derived MSC; SMSC: synovial MSC.

### Preparation and modification of EVs

The important role of EVs in the diagnosis, prognosis, and regeneration of diseases promotes the development of EV isolation techniques. As EVs are nanoparticles and originate from a complex fluid environment, obtaining homogeneous and high purity therapeutic EVs remains a great challenge. The current methods for isolating EVs are mainly based on physical (size and density) and chemical (affinity) properties, as well as immunoaffinity chromatography (combining the use of liquid chromatography with the specific binding of antibodies)^[58-60]^. However, the most ubiquitous adopted method for EV preparation is still dominated by ultracentrifugation (UC). Methods based on micromachining technology, due to label-free processing, cost-effectiveness, and amenability to automation, have emerged as a promising method for label-free EV separation. Inglis *et al.*^[61]^ designed and implemented theoretical models for the critical particle size of fractionation in deterministic lateral displacement (DLD) separation arrays, aiming to provide a theory and experimental measurements for critical conditions. Wunsch *et al.*^[62] ^applied this technique to the true nanoscales, where they could function in EV separation, such as exosomes. This study revealed a potential for the on-chip sorting of these nanoparticles. For fast EV enrichment, a technology that integrates 1024 nanoscale DLD (nano-DLD) arrays on a single chip allows parallel processing to reach 900 μL/h^[63]^. Moreover, compared with other methods, including UC, UC plus density gradient, size-exclusion chromatography, and co-precipitation, the chip showed a superior efficiency. Recently, our team reported a novel exosome detection method via the ultrafast-isolation system (EXODUS) that allowed automated label-free purification of exosomes from various biofluids^[64]^. We also reported a size-based EV isolation tool, namely ExoTIC, to efficiently isolate EVs from small sample volumes, providing an analytical tool for preclinical studies^[65]^. These techniques are advantageous for the standardized preparation of MSC-EVs and can accelerate the clinical translation of MSC-EVs.

The massive production of MSC-EVs is another research hotspot for the cell-free treatment model. To expand the clinical translation of MSC-EVs, the methods used for large-scale production of EVs with a good manufacturing practice (GMP) level are necessary. To date, the efficiency of EVs has been improved by changing the culture method of donor cells, such as three-dimensional (3D) environmental culture^[66-68]^. Natural extracellular matrix and 3D biological scaffolds were used for cell attachment, cell growth, and production of functional EVs^[69]^. Using the 3D spheroid culture method based on photolithography and micro-pattern technologies, gene expression profiles of MSCs were confirmed with a high differentiation efficiency^[70]^. Cone *et al.*^[71] ^assessed the potential therapeutic effects of EVs from a 3D culture of bone marrow-derived MSCs (BMSCs) in an Alzheimer’s disease (AD) model, and it was revealed that intranasally administration of MSC-EVs ameliorated pathology and cognitive deficits of AD. Mend *et al.*^[72]^ reported a bioreactor-based and clinical-grade production of engineered exosomes with the ability to target oncogenic KRAS. The clinical-grade product was tested in multiple *in vitro *and* in vivo* experiments to confirm the feasibility of various therapies for human diseases.

In addition to natural EV agents, the development of different modifications of MSC-EVs may provide new approaches for gene therapy and drug delivery. Exogenous nucleic acids, such as miRNA, siRNA, DNA carrier, and DNA probe, are loaded into EVs by electroporation, accompanied with favorable biocompatibility and biostability. For instance, the engineered MSC-EVs can serve as a promising anti-osteoporosis therapy via loading Shn3 gene-targeted siRNA^[73]^. Angiopep-2 (Ang) is a ligand that binds specifically to the lipoprotein receptor-related protein 1 receptor and improves the high efficiency of transport across the blood-brain barrier (BBB)^[74-76]^. Several scholars designed a multifunctional exosome-mimetics decorated with Ang and load docetaxel for anti-glioblastoma therapy^[77]^. This personalized approach also achieved the purpose of targeted therapy.

Overall, with the advances of nanomedicine in molecular cell biology, pharmaceutical science, and nano-engineering^[78]^, higher requirements for engineering transformation of MSC-EVs are demanded, especially standardized production and storage of MSC-EVs.

### EV-based therapeutics

**Figure 2:**
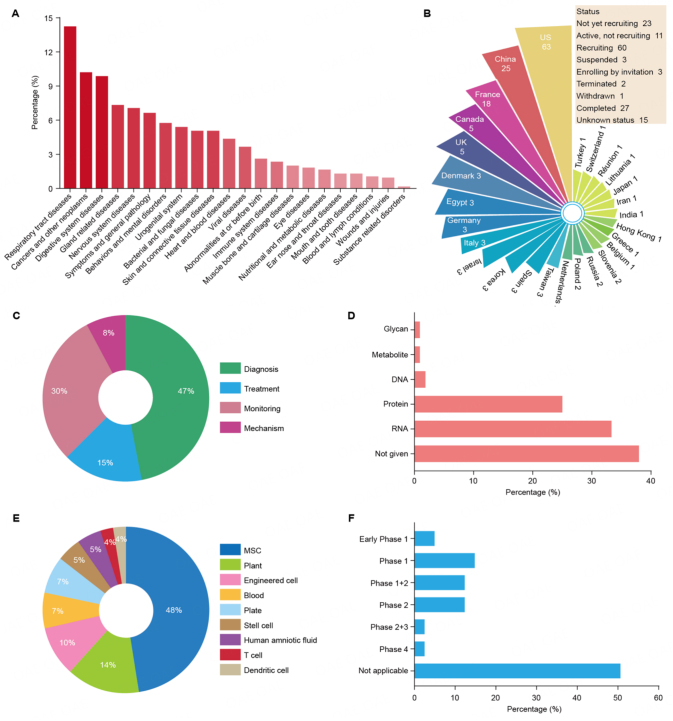
The progress of 143 clinical trials involving EVs worldwide [www.clinicaltrials.gov (Accessed: August 2021)]. (A) The number of studies on EVs by physiological structure. There are 22 categories according to the body structure in 143 studies. (B) The number of studies on EVs is classified according to country and project progress status. According to country classification, 27 countries participated in the 143 studies on exosomes, and there are nine research statuses in 143 studies. (C) The percentage is presented based on the research purpose. There are four different directions of EV-based research, including diagnosis, treatment, monitoring, and mechanism. (D) The contents of EVs were evaluated in 108 studies. Others were used to illustrate an unclear description. (E) The proportion of cell sources used for the treatment with EVs in 42 studies. There are nine therapeutic sources of EVs, mainly originating from MSCs. (F) According to the definition presented by the US Food and Drug Administration (FDA), the number of studies in each clinical trial stage is shown. “Not Applicable” is used to describe trials without FDA-defined phases. EVs: Extracellular vesicles; MSCs: mesenchymal stem cells.

EVs have been extensively studied in clinical trials. A statistical analysis of 143 EV-dependent clinical trials was performed, and significant conclusions were obtained, as shown in Figure 2 [www.clinicaltrials.gov (Accessed: August 2021)]. The majority of clinical studies are conducted in the United States and China, and respiratory, tumor, and gland-related diseases were research hotspots [Figure 2A and B]. Based on research purposes, we divide all studies into four groups, which are followed by diagnosis, monitoring, treatment, and mechanisms [Figure 2C]. Then, we calculate the percentages of EVs involved in 108 studies that are mainly related to exosomal RNAs and proteins [Figure 2D]. Treatment-related research accounted for 15% of all items, which are mostly derived from MSCs [Figure 2E]. As shown in Figure 2F, most clinical trials are still in the infancy stage. At present, therapeutic vesicles are widely used in cardiovascular and cerebrovascular diseases, respiratory diseases, neurological diseases, cancer, and bone regeneration by affecting cell cycle arrest or apoptosis^[79-84]^. As a good example of the application of EVs in bone regeneration, osteoarthritis (OA) is a joint degenerative disease characterized by synovial inflammation and articular cartilage damage. The treatment of OA mainly depends on surgery and drugs. Several studies have shown that EVs maintain homeostasis and improve the severity of osteoarthritis pathologically through local and distal intercellular and intracellular signaling pathways^[84-86]^. In a rat model of glucocorticoid-induced femoral head necrosis, human umbilical cord-derived MSC-EVs (UMSC-EVs) could reduce the apoptosis of bone cells through the miR-21-PTEN-AKT signaling pathway^[87]^.

## MSC-EV-BASED THERAPY FOR OPHTHALMIC DISEASES

### Corneal disease

Corneal disease is the major cause of vision loss, which may be caused by several clinical conditions, including physical trauma, chemical burns, infections, limbal stem cell defects, age-related degeneration, and corneal malnutrition^[26]^. Although corneal transplantation has made significant progress in the past decade, there are still problems, such as few donors, immune rejection, and long-term use of immunosuppressant agents^[88,89]^. The role of MSCs in corneal regeneration therapy can be directly attributed to cell replacement^[90]^ and delivering targeted biomolecules^[91-94]^. Several scholars attempted to incorporate hydrogel with exogenous recombinant human stromal cell-derived factor-1 alpha for corneal epithelial regeneration^[95]^. EVs have a promising prospect of therapeutic applications, as they inherit parental cell-derived molecules. Thus, MSC-EVs have also been applied in the therapy of corneal disease.

Many studies have confirmed the therapeutic efficiency of MSC-EVs for eye diseases including corneal and retinal models *in vitro *and* in vivo*, as presented in Table 2. Overall, tissue sources of MSCs for ocular diseases mainly originate from human corneal stroma, bone marrow, umbilical cord, and adipose tissues. In-depth analysis and generalization of the mechanism of MSC-EVs for corneal disease can be summarized in the following aspects: (1) MSC-EVs enhance the proliferation of human corneal epithelial cells (HCECs) and promote the migration of HCECs after corneal disease^[96-98]^; (2) MSC-EVs reduce scar formation, neovascularization, and hemorrhage after corneal disease^[96,97,99]^; (3) MSC-derived products decrease the levels of inflammatory cytokines, such as interleukin (IL)-1β, IL-6, and IL-10^[98]^; (4) MSC-EV-based treatment can inhibit neutrophil infiltration and polarize M2 macrophage infiltration^[98,100]^; and (5) MSC-EVs depress the expression levels of fibrotic genes (Col3a1 and Acta2) and serve as a delivery vehicle for miRNA in blocking corneal scarring blocking scarring and initiating regeneration after wound healing^[100]^.

**Table 2:** Studies on the therapy of ocular diseases using MSC-EVs

**Position**	**EV source**	**Administration route/dose**	**Results**	**Effector molecule**
Cornea	BMSCs	Viscoelastic gel carrier/unclear	Enhance HCECs proliferation and wound healing; reduce scar formation, neovascularization, and hemorrhage	Unclear^[96]^
	BMSCs	Co-culture/unclear	Induce proliferation and migration of damaged HCECs; inhibit cell apoptosis	Unclear^[97]^
	ADSCs	Topical administration/unclear	Promote proliferation and migration of HCECs, reduce inflammatory cytokine levels, polarize infiltrating macrophages toward M2	Unclear^[98]^
	CSSCs	EVs drop/5.0 × 10^6^ particles	Accelerate wound healing	Unclear^[99]^
	CSSCs	Topical fifibrin gel/1 × 10^7^ particles	Decreased expression of fibrotic genes Col3a1 and Acta2, blocked neutrophil infiltration	miRNA^[100]^
	ADSCs	Co-culture/1.61 × 10^10^ particles	Toxicological testing	Unclear^[101]^
	BMSCs	Co-culture/unclear	facilitate wound healing	Unclear^[102]^
Retina	UMSCs	IV/2.5 μg	Inhibition of MCP-1	MCP-1^[28]^
	BMSCs	IV/3 × 10^9^ particles	Through miRNA dependent mechanisms	miRNA^[56]^
	UMSCs	Tail vein/55 μg	MiR-126 expression and downregulating the HMGB1 signaling pathway	miR-126^[103]^
	ADSCs	IS/unclear	Delivering microRNA-222 acts as mediators in retinal tissue repair	miRNA-222^[104]^
	BMSCs	IV/4 × 10^9^ particles	Reduce neuroinflammation and neuronal apoptosis	Unclear^[105]^
	BMSCs	Tail vein/30 μg	Inhibit activation of antigen-presenting cells and suppress the development of Th1 and Th17 cells	Unclear^[106]^
	UMSCs	IV/0.05 μg	Ameliorate retinal injury via downregulation of VEGF-A	Unclear^[107]^
	UMSCs	IV/1 × 10^9^ particles	Promoting the RGCs survival and glia cells activation	Unclear^[108]^
	BMSCs	IV/1 × 10^9^ particles	Preserving RGC numbers and protecting against axonal degeneration	Unclear^[109]^
	ES-MSCs	IO/15 μg	Improved Brn3a+ RGCs survival and improved cognitive visual behavior	Unclear^[110]^

MSC-EVs: Mesenchymal stem cells-derived extracellular vesicles; BMSCs: bone marrow-derived MSCs; HCECs: human corneal epithelial cells; ADSCs: adipose tissue-derived mesenchymal stem cells; CSSCs: corneal stromal stem cells; IV: intravitreal injection; UMSCs: umbilical cord-derived MSCs; IS: intravenous subconjunctival; RGCs: retinal ganglion cells; ES-MSCs: embryonic stem cell-derived MSCs; MCP-1: monocyte chemoattractant protein-1; HMGB1: high mobility group 1; IO: intravenous.

EV-encapsulated natural lipid bilayers were considered as a good carrier to protect miRNAs from degradation. The differences in certain miRNA or miRNA expressions of EVs showed the diversity of receptor phenotypic regulation by non-coding RNA^[111-113]^. To elucidate the molecular mechanisms of EVs in the treatment of corneal wounds, researchers have performed numerous cutting-edge experiments. Using small-interfering RNAs (siRNAs) to knock down the mRNA of ESCRT protein Alix resulted in a reduction of 85% of EV miRNA; thus, EVs lacking miRNA lost their regeneration function^[100]^. The finding indicates that miRNA is a key adjustable molecule for EVs to exert restorative effects. Some studies have concentrated on the exosomal miRNA functions (i.e., regulating angiogenesis and anti-fibrotic immunosuppressive agents)^[114,115]^, suggesting that miRNAs play an important role in maintaining homeostasis. Moreover, the miRNA expression profiles are variable in different cell types, which were mainly reflected in the number and category^[116]^.

The therapeutic potential of MSCs can be related to cultivation conditions and cellular microenvironment. The influences of two-dimensional (2D) and 3D culture conditions on the therapeutic efficacy of MSC secretomes on corneal wound healing were studied with *in vitro* cell and organ culture experiments^[84]^. Notably, the secretomes from the MSC 3D environment facilitate wound healing in corneal fibroblast cells and enhance epithelialization. Ha *et al.*^[101] ^conducted a toxicological evaluation of exosomes derived from human adipose tissue-derived mesenchymal stem cells (ADSCs), and the eye irritation test suggested that ADSC exosomes are safely used as a topical treatment.

Altogether, MSC-EV-dependent therapeutic molecules can regulate intercellular signaling pathways, and engineered EVs may be an emerging agent for corneal diseases. 

### Retinal diseases

Retinal ganglion cell (RGC) death is the irreversible endpoint of optic neuropathy. Glaucoma is a group of progressive optic neuropathies characterized by the gradual disappearance of RGCs. Under an *in vitro* strict culture, MSCs can induce differentiation into neuroectodermal cells, including neuronal cells^[117]^. MSCs used for the treatment of glaucoma mainly contribute to producing neurotrophins, differentiation into functional RGCs, and interaction with TM (trabecular meshwork), thereby reducing the intraocular pressure of glaucoma^[118]^. The capacity of MSC-EVs for neuroprotection and immunomodulation in the treatment of retinal diseases is mainly due to miRNA-dependent and inflammatory responses^[119]^.

Studies have proven that miR-34a-5p, miR-126, and miR-222 affect the progression of retinal damage through diverse mechanisms^[103,104,120]^. For instance, in a cell model of diabetic retinopathy, MSC-derived exosomal lncRNA SNHG7 suppresses endothelial-mesenchymal transition and tube formation by negatively regulating miRNA^[120]^. miR-126 has been reported as an endothelial cell-restricted miRNA that mediates inflammation and vascular development^[103]^. HMGB1, one of the target genes of miR-126, has high expression levels in various inflammatory and autoimmune diseases^[121]^. Co-culture of MSC-EVs with high expressions of miR-126 and human retinal endothelial cells was found to significantly reduce the level of HMGB1 protein and improve retinal inflammation caused by hyperglycemia in diabetic rats^[103]^. A previous study showed that MSC-EVs are endocytosed by retinal neurons, retinal ganglion cells, and microglia as biomaterials for neuroprotective and regenerative therapy of retinal disorders^[105]^. Moreover, in a clinical trial, Zhang *et al.*^[122]^ also proved that MSC-EV therapy may be an advantageous and safe method for improving visual outcomes after surgery for refractory macular holes.

With the advent of induced pluripotent stem cells (iPSCs), tremendous progress has been made in stem cell biology and regenerative medicine. Human iPSCs are widely used in animal modeling, drug discovery, and cell therapy development^[123]^. Besides MSC-EV-based therapies, iPSCs can also be used as an unlimited source for retinal degenerative diseases^[124]^. Retinal pigment epithelial cells derived from iPS (iPS-RPE) replace damaged or diseased cells and promote the healing and repairing of eye tissues^[125]^. Studies have shown that iPS-RPE plays an effective role in delaying photoreceptor degeneration by stably surviving in a degraded ocular environment and releasing neuroprotective factors, such as the pigment epithelium-derived factor^[126]^. To obtain an adequate source of cells, Reichman *et al.*^[127]^ developed a two-step culture system to effectively differentiate iPSCs into retinal cells and achieved large-scale production and storage of hiPSCs-derived retinal cells and tissues. The development of iPSCs is expected to be another novel approach to treat retinal diseases in the future.

### Other ocular diseases

The immunoregulatory effects of MSC-EVs have been reported in a variety of experimental models, such as rheumatoid arthritis^[128]^, neurodegenerative disorders^[129]^, and inflammatory bowel disease^[130]^. Studies have demonstrated that the anti-inflammatory effect of MSC-EVs is closely associated with regulating the activity of macrophages^[131-133]^, natural killer cells^[134]^, B cells^[135,136]^, and T cells^[137,138]^. Scholars also used this positive influence in the modeling of inflammatory-related eye diseases. Uveitis, an inflammatory disorder involving the pigmented vascular coat of the eyeball, can result in blindness in the absence of timely therapy. Similar to inflammatory eye disease, MSC-EVs suppress autoimmunity in models of experimental autoimmune uveoretinitis (EAU) by inhibiting the development of T cells^[106]^. Administering MSC into rodents with induced models of clinical diseases with an appropriate dose can result in the reversal of abnormalities for weeks thereafter. Zhang *et al.*^[139]^ examined the long-term effects of BMSCs in a recurrent EAU model in rats. The results demonstrate that BMSCs significantly decreased responses of T helper 1 (Th1) and Th17 cells, suppressed the functions of antigen-presenting cells, and upregulated T regulatory cells. In the study of EAU in Lewis rats, MSCs showed an inhibitory effect on activation and maturation of dendritic cells via regulation of STAT1 and STAT6 phosphorylation^[140]^. In the subsequent studies using MSC-EVs on the same EAU models, it was found that administration of MSC-EVs could ameliorate uveitis similar to their parent cells^[141]^. Using *in vitro *experiments, the effects of MSC-EVs on immune-cell activation were assessed using allogeneic mixed lymphocyte reaction assays. Consistent with previous MSC-related findings, MSC-EVs simultaneously reduce the infiltration of T cells and the levels of inflammatory cytokines^[106]^.

Sjögren’s syndrome (SS), a chronic multi-system autoimmune disease mainly involving the exocrine gland, causes dry mouth (hyposialia or even asialia) and dry eye (xerophthalmia)^[142,143]^. MSCs, as a therapeutic approach to treat SS, have been assessed in preclinical trials^[144,145]^. In an *in vivo *study, Xu *et al.*^[146] ^proposed a novel therapeutic approach to alleviate diseases in patients with primary SS by infusing allogeneic UMSCs. These effects are nutritive, anti-inflammatory, anti-immunologic, and associated with the healing of abnormalities. Regarding EVs secreted by MSCs, MSC-EVs may be an ideal replacement for decreasing the pathogenesis of SS. Rui *et al.*^[147] ^found that murine olfactory ecto-MSC-derived exosomes significantly improved impaired immunosuppressive function of myeloid-derived suppressor cells by administering MSC-EVs intravenously into mice with induced models of SS. Considering the limited expandability, significant donor variations, and safety concerns of MSC sources, it is essential to optimize a protocol that can be easily scaled up to produce standardized iPSC-MSCs, showing the same potential to prevent the progression of SS^[148]^.

Taken together, a combination of MSC and MSC-EVs with emerging technologies may provide novel insight for into the therapy of eye diseases.

## CHALLENGES AND PROSPECTS

Although MSC-EVs are regarded as a new treatment strategy, their affiliated clinical challenges are worthy of further assessment.

Firstly, obtaining an appropriate cell line is a prerequisite for collecting EVs. The existing research on eye diseases is summarized in Table 2. Therapeutic vesicles are mainly sourced from cells of adipose, bone marrow, umbilical cord, and cornea. However, there is a lack of research comparing MSCs from various sources for the treatment of eye diseases. The impurity of MSCs leads to the complexity of the contents of EVs, negatively influencing their performance. Therefore, to obtain high-quality products, the tissue source, identification, and functional testing of MSCs are required. Despite that, the mechanism of MSC-EVs for other diseases has been studied in detail [Table 1], EV contents such as protein and genetic cargo for the therapy of eye diseases remain unknown. Most studies still observe the curative effect by injecting intact MSC-EVs into model animals [Table 2]. Above all, the personalized design of EVs is also essential; to date, based on the pathogenesis and pathological process, various genetic or non-genetic engineering methods have been developed for producing EVs with specific biological characteristics^[148-152]^. To achieve precision treatment, it is necessary to master the knowledge of EVs related to the composition, identification, purification, and function of distinct cell origins and under various physiological statuses.

Another question is how to achieve a standardized and stable production of EVs as drugs with a GMP level. Studies showed that the heterogeneity of EVs derives from their size, contents, and cell origin^[153]^. Proteomics analysis of EVs revealed the heterogeneity of protein profiles, suggesting that there is an urgent need to optimize and standardize the purification method to obtain high-quality EVs^[154]^. Despite the emergence of many novel EV preparation techniques, a consensus on manufacturing the therapeutic vesicles from cell culture needs to be reached. Referring to the recent ISEV workshop position papers, there are some issues that should be considered^[155]^. First, the maximum cell death rate must be less than 5% to prevent dead cells from releasing particles unrelated to the therapeutic purpose that affect EV purity. Secondly, the detection of cellular microbial contamination such as mycoplasma and viruses must be performed to meet the requirements of standard level for clinical use. In addition, the standardized protocol of MSC-EV preparation from cultured cell-conditioned-medium should be automated, timesaving, and have a high recovery efficiency. Some operational considerations need to be noted, for example, avoiding repeated freeze–thaw samples and ensuring temperature control during EV separation to prevent the destruction of functional molecules in vesicles. The EXODUS platform is a promising tool that highly satisfies all demands for the collection of EVs in a large-volume culture medium^[64]^. Finally, the quality of the MSC-EV preparation should be evaluated by the size, morphology, specific markers, and detection of contaminants. The scientific storage and transportation conditions of MSC-EVs are important to ensure the efficacy.

Given the security of EVs, the mechanism-dependent and safety data of MSC-EVs mainly originate from preclinical *in vitro *and animal research. However, it is important to indicate whether the results of the application of MSC-EVs in animal experiments can be reliably used in human clinical trials. This relies on conducting a large number of clinical trials. For the treatment of eye diseases, the administration routes of EVs mainly involve injection, eye drops, and dressing. MSC-EVs serve as an ideal source for drug delivery, regardless of encapsulating in biomaterials or dissolving in liquid^[156]^, maintaining the biological activity.

## CONCLUSIONS

The main limitation of MSC therapy for optic neuritis is the difficulty of reaching the site of pathology in the optic nerve and retina. In the view that EVs can cross the BBB, while MSCs cannot, and deliver various therapeutic factors to the brain, MSC-EVs have been extensively tested as a beneficial treatment for the control of chronic inflammation of the central nervous system. Adequate EVs can be produced by large-scale expanding parental cells. For precision medicine, engineering and modification of EVs can improve the targeted drug delivery efficiency by overexpressing therapeutic molecules, such as miRNAs. Although additional advanced research is still required to explore the mechanism of EVs in the therapy of various eye diseases, it is undeniable that MSC-EVs have promising prospects in ocular repairing.

## DECLARATIONS

### Copyright

© The Author(s) 2022.
